# Redescription of the poorly known Central Asian *Titanoeca lehtineni* Fet, 1986 (Araneae, Titanoecidae)

**DOI:** 10.3897/zookeys.144.2266

**Published:** 2011-11-03

**Authors:** Mikhail M. Omelko

**Affiliations:** 1 Far Eastern Federal University, Sukhanova, 8, Vladivostok 690950 Russia and Gornotaezhnaya Station FEB RAS, Gornotaezhnoe Vil., Ussuriyski Dist., Primorski Krai 692533 Russia

**Keywords:** *Titanoeca*, Turkmenistan, redescription, spider

## Abstract

A poorly known spiders species, *Titanoeca lehtineni* Fet, 1986, from southwestern Central Asia is redescribed and diagnosed.

## Introduction

*Titanoeca lehtineni* was described on the basis of 92♂ and 4♀ specimens from seven localities collected in south Turkmenistan (Fet 1986). Besides Turkmenistan *Titanoeca lehtineni *has been reported from southern Kazakhstan (Zyuzin and Tarabaev 1994; Zyuzin et al. 1995). The original description was short and included only three figures: two figures of the male palp and one of the epigyne. The vulva and details of the bulbus as well as complex tibial apophysis were not depicted. For this reason, and because the original description was in Russian, the species is more completely redescribed here in a more accessible format.

## Materials and methods

Specimens were photographed using an Olympus Camedia E-520 camera attached to an Olympus SZX16 stereomicroscope at the Zoological Museum, University of Turku. Photographs were taken in dishes with paraffin on the bottom. Images were montaged using “CombineZP” image stacking software. Different sized holes were made in the paraffin to keep the specimens in the correct position. All measurements are in mm. The label is spelled like in the original, although some names and administrative units names have changed. Specimens are deposited in the Zoological Museum of the Moscow State University (ZMMU) and in the Siberian Zoological Museum (SZM) in Novosibirsk.

## Taxonomy

### Titanoeca lehtineni

Fet, 1986

http://species-id.net/wiki/Titanoeca_lehtineni

Figs 1- 10

Titanoeca lehtineni Fet, 1986: 65, f. 1–3 (♂♀).

#### Material examined.

TURKMENISTAN: 2♂ 16♀ (ZMMU), Mary Area, Kushka (now = Serhetabad) Distr., ca 18 km S of Kyzyldzhar kordon (=field station), ca 1 km ESE of Eroilanduz Salt Lake, 375 m, 35°39'40"N, 61°50'13"E, 7.04.2002 (A.V.Gromov); 1♂ (SZM), Kopetdagh Reserve, Kalinskiy Zakaznik, 18.05.1987 (V.Dubatolov).

#### Note.

According to the original description the types were deposited in the Zoological Institute in Sankt Petersburg (ZISP) and in the Zoological Museum of the Moscow State University (ZMMU). Paratypes in ZMMU were not found (Mikhailov, personal communication) and were not searched for in ZISP. I am sure about the current identification because the material studied was collected from within the same area that the type series originated.

#### Diagnosis.

This species can be easily recognized from all other congeners and even all species of Titanoecidae by its very long embolus which forms several coils (Figs 2, 5), and the weakly sclerotized epigyne (Fig. 9) with extremely long insemination ducts (Figs 6–8, 10).

#### Description.

Total length (♂/♀) 3.25–3.5/3.5–4.6. Carapace: 1.45/1.68 long, 1.13/1.23 wide. Males (Fig. 4) and females uniformly brown, without any pattern. Tibiae and metatarsi in male with ventral spines (Fig. 4).

**Figures 1–5.**
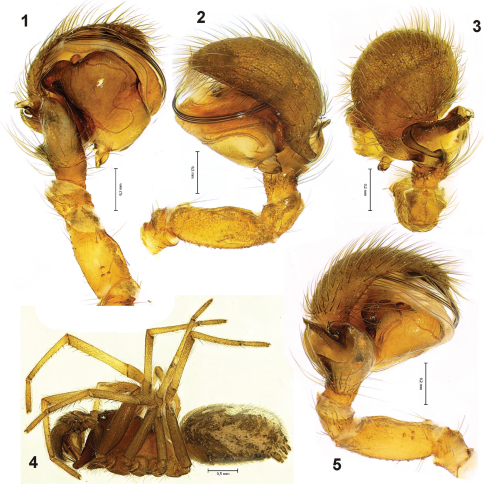
Male of *Titanoeca lehtineni*. **1** palp, ventral **2** palp, retrolateral **3** palp, dorsal **4** habitus, lateral **5** palp, prolateral.

Palp as in Figs 1–3, 5. Femur shorter than cymbial length and even shorter than tegulum (Fig. 2). Tibial apophysis large on dorsal and prolateral sides. Tegulum wider than long (Fig. 1), embolus well visible in ventral, pro- and retrolateral views.

**Figures 6–10.**
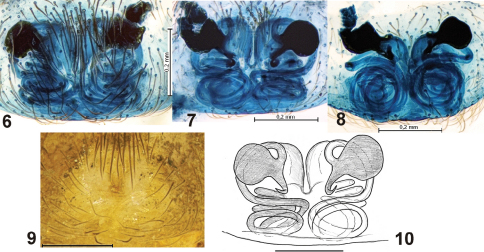
Epigyne of *Titanoeca lehtineni*.** 6, 9** ventral view **7, 10** dorsal view **8** caudal view. **6–8, 10** after maceration. Left receptaculum was broken during maceration.

Epigyne as in Figs 6–10. Epigyne is weakly sclerotized and has no distinct margins. It is also lighter than other parts of the abdomen. Therefore, the adult female could easily be mistaken for a juvenile specimen. Vulva complicated, with relatively small round receptacula in the anterior part, long insemination ducts forming at least seven coils and strongly sclerotized fertilization ducts.

#### Comments.

The specimens examined are slightly smaller than the specimen measured by Fet (1986): 3.75/5.0 total length, 1.75/2.0 long, 1.38/1.0 wide. It seems that the width of the female carapace was measured incorrectly in the original description. None of Titanoecidae have the carapace twice as long as it is wide. Although the type series of this species is fairly large, variation in size was not mentioned by Fet (1986).

This species has exceptionally long insemination ducts compared to other species in this family. They appear to correspond to the very long embolus in the male. Insemination ducts in *Titanoeca lehtineni* are longer than in *Nurscia sequerai* (Simon, 1892) (cf. fig. 6  in Hubert 1966). *Titanoeca lehtineni* also has an unusual position of the receptacula in the anterior part of epigyne, far from epigastric furrow.

#### Distribution.

So far this species is known from southern and southwestern Turkmenistan and southwest Kazakhstan. Its occurrence in western Uzbekistan and northeastern Iran is highly likely.

**Table 1.** Length of leg segments in male and female (♂/♀)

	Femur	Patella	Tibia	Metatarsus	Tarsus	Total
I	1.35/1.33	0.52/0.60	1.17/0.95	1.05/0.90	0.58/0.55	4.67/4.33
II	1.13/1.13	0.43/0.50	0.83/0.75	0.85/0.75	0.50/0.50	3.74/3.63
III	0.97/1.05	0.38/0.45	0.70/0.65	0.73/0.76	0.48/0.40	3.26/3.31
IV	1.17/1.25	0.43/0.53	0.97/1.0	0.88/0.87	0.45/0.43	3.90/4.08

## Supplementary Material

XML Treatment for Titanoeca lehtineni
